# Additional Burden of Diseases Associated with Cadmium Exposure: A Case Study of Cadmium Contaminated Rice Fields in Mae Sot District, Tak Province, Thailand

**DOI:** 10.3390/ijerph120809199

**Published:** 2015-08-07

**Authors:** Nisarat Songprasert, Thitiporn Sukaew, Khanitta Kusreesakul, Witaya Swaddiwudhipong, Chantana Padungtod, Kanitta Bundhamcharoen

**Affiliations:** 1 International Health Policy Program, Ministry of Public Health, Nonthaburi 11000, Thailand; E-Mails: nisarat@ihpp.thaigov.net (N.S.); thitiporn@ihpp.thaigov.net (T.S.); khanitta@ihpp.thaigv.net (K.K.); 2 Department of Community and Social Medicine, Mae Sot General Hospital, Tak 63110, Thailand; E-Mail: swaddi@hotmail.com; 3 Bureau of Occupational and Environmental Disease, Department of Disease Control, Ministry of Public Health, Nonthaburi 11000, Thailand; E-Mail: cpadungt@gmail.com

**Keywords:** cadmium, Mae Sot, DALYs, disability adjusted life years

## Abstract

The cadmium (Cd) contaminated rice fields in Mae Sot District, Tak Province, Thailand has been one of the major environmental problems in Thailand for the last 10 years. We used disability adjusted life years (DALYs) to estimate the burden of disease attributable to Cd in terms of additional DALYs of Mae Sot residents. Cd exposure data included Cd and β_2_–microglobulin (β_2_-MG) in urine (as an internal exposure dose) and estimated cadmium daily intake (as an external exposure dose). Compared to the general Thai population, Mae Sot residents gained 10%–86% DALYs from nephrosis/nephritis, heart diseases, osteoporosis and cancer depending on their Cd exposure type and exposure level. The results for urinary Cd and dietary Cd intake varied according to the studies used for risk estimation. The ceiling effect was observed in results using dietary Cd intake because of the high Cd content in rice grown in the Mae Sot area. The results from β_2_-MG were more robust with additional DALYs ranging from 36%–86% for heart failure, cerebral infarction, and nephrosis/nephritis. Additional DALYs is a useful approach for assessing the magnitude of environmental Cd exposure. The Mae Sot population lost more healthy life compared to populations living in a non- or less Cd polluted area. This method should be applicable to various types of environmental contamination problems if exposure assessment information is available.

## 1. Introduction

Cadmium is a highly toxic metal with a slow elimination rate; its half-life in the human body ranges between 10–30 years. Chronic exposure to low-level cadmium is associated with a number of health outcomes, such as end-stage renal failure, early onset of diabetes, renal complications, osteoporosis, disrupted blood pressure regulation, and increased cancer risk [1,2,3]. Exposure to cadmium can be measured in blood for acute exposure and in urine for chronic exposure and body burden [2,4]. An early renal effect from cadmium is excretion of tubular proteinuria such as β_2_–microglobulin (β_2_-MG), retinol binding protein, α_1_-microglobulin, and enzymes [1,5]. Using the World Health Organization (WHO) standard, the reference levels of urinary cadmium (U-Cd) are 2 µg/g creatinine for environmental exposure, 5 µg/g creatinine for occupational exposure, and 10 µg/g creatinine for possible renal damage caused by cadmium [6] and the level for urinary β_2_-MG is below 300 µg/g creatinine [7].

In the Mae Sot District, Tak Province, northwestern Thailand, the paddy fields received irrigation from the two creeks (Mae Tao and Mae Ku) passing through a zinc rich area where the zinc mine had been actively operated for more than 20 years. From the surveys from 2000 to 2004, over 90% of the grown rice grain samples in the cadmium-contaminated area contained cadmium content above the maximum permissible level of 0.4 mg/kg [8]. In 2004 and 2009, a population screening survey for cadmium exposure using U-Cd measurement was first and second conducted among ~7600 and ~6700 residents, respectively, aged 15 years and older living in these contaminated villages [9,10,11]. In both surveys, prevalence of high urinary cadmium associated with consumption of locally grown rice was greater than that for tobacco smoking [9]. Importantly, many studies in this cadmium-contaminated area have shown positive relationships between urinary cadmium and renal dysfunction, bone toxic effects, hypertension and urinary stone disease [12,13,14,15,16,17,18,19]. A health risk management plan for the cadmium-contaminated area was conducted. The government purchased all rice grown in these contaminated areas and supported the production of non-food crops such as sugar cane, decorative palm and rubber plantation to replace rice cultivation [20]. However, it has not been fully settled on whether and how the contamination could be cleaned up. In 2015, the cadmium contamination problem in Mae Sot District remains unresolved and the search for alternative ways to manage health outcomes more economically, socially, and culturally is ongoing. The objective of this study was to provide quantitative evidence pointing at the magnitude of the cadmium contamination in Mae Sot on population health. We presented the impact of the loss of population health through a modified version of a disability-adjusted life year (DALY), a summary measure of population health representing a combination of life years lost (or gained) and time spent with disease, adjusted for the severity of that particular disease in a single indicator [21,22,23,24]. Instead of using DALYs, we calculated a modified version of DALYs called additional DALYs (add-DALYs) because of cadmium exposure for the population in the contaminated area of Mae Sot District.

## 2. Materials and Methods

We estimated add-DALYs attributable to cadmium exposure of the population in the contaminated area of Mae Sot District based on our cadmium exposure data. The study protocol was approved by the Mae Sot Hospital Ethical Committee. The details of the studies and sources of information used for parameters estimation are described below. 

### 2.1. The Cadmium Contaminated Area

Mae Sot is a district in Tak Province situated in the north of Thailand. The cadmium contaminated areas covered rice fields irrigated with water from the Mae Tao and Mae Ku creeks running through a zinc mine and included 12 villages in three sub-districts: Mae Tao (6 villages), Mae Ku (3 villages) and Pra Tad Pha Dang (3 villages). The term “Mae Sot area” was used to represent these sub-districts in this study.

### 2.2. Cadmium Exposure Data of Population in Mae Sot Area

**Figure 1:**
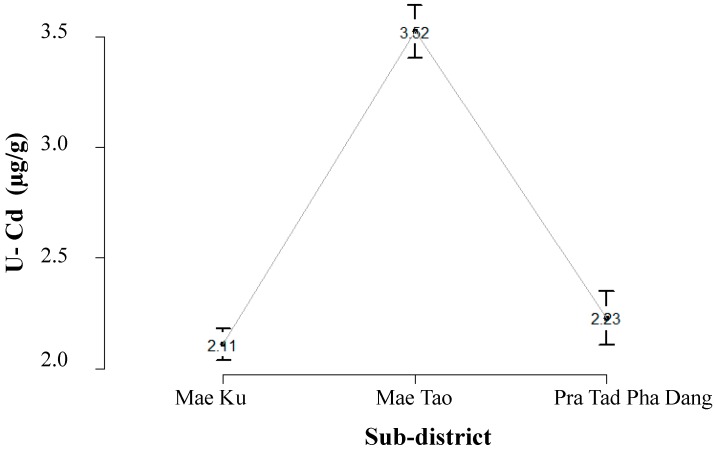
Distribution of urinary cadmium (U-Cd) in the surveyed population in Mae Sot areas: residents in Mae Ku (mean = 2.11 µg/g creatinine, 95% CI = 2.04–2.18), Mae Tao (mean = 3.52 µg/g creatinine, 95% CI = 3.41–3.64), and Pra Tad Pha Dang (mean = 2.23 µg/g creatinine, 95% CI = 2.11–2.35).

There were two types of cadmium exposure data used in this study: Cd content and β_2_-MG in urine (as an internal exposure dose) and estimated cadmium daily intake via Cd-contaminated rice ingestion (as an external exposure dose). Urinary cadmium concentration was collected from 6642 of 9411 subjects (70%) aged ≥15 years old from 12 villages in Mae Sot area to represent the distribution of internal cadmium exposure of Mae Sot population. This information was obtained from a screening survey conducted by the Department of Community and Social Medicine, Mae Sot General Hospital in 2009 [9]. Figure 1 shows the population distribution of U-Cd across different sub-districts in the study: from Mae Ku (*n* = 2363), Mae Tao (*n* = 2900), and Phra Tad Pha Dang (*n* = 1379). Approximately 50% of the total population had U-Cd over the standard limit of 2 µg/g creatinine.

**Figure 2:**
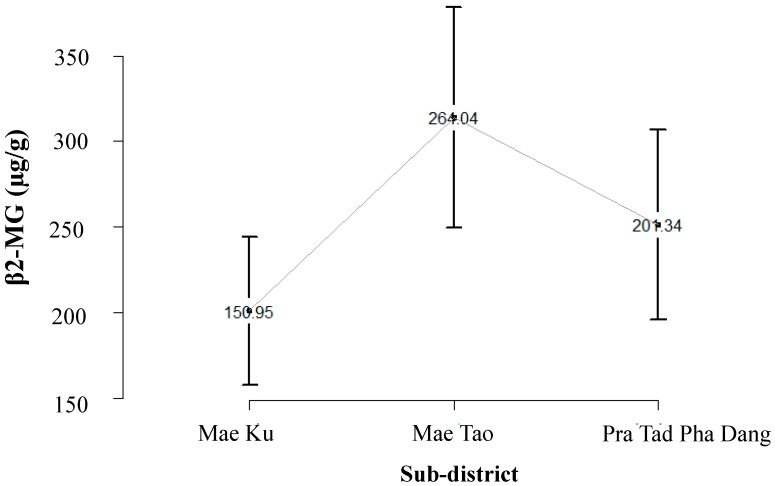
Distribution of β_2_–microglobulin (β_2_-MG) in surveyed population in Mae Sot areas: residents in Mae Ku (mea*n* = 150 µg/g creatinine, 95% CI = 108–194), Mae Tao (mean = 264 µg/g creatinine, 95% CI = 199–328), and Pra Tad Pha Dang (mean = 201 µg/g creatinine, 95% CI = 146–257).

The β_2_-MG data was obtained from the subset of U-Cd survey in 2004 [13], which included 480 residents with U-Cd exceeding 5 µg/g creatinine. Figure 2 shows the distribution of β_2_-MG level across the population in the study: from Mae Ku (*n* = 75), Mae Tao (*n* = 283) and Phra Tad Pha Dang (*n* = 122). The body burden of β_2_-MG in these inhabitants ranged from 2.17–146,129 µg/g creatinine, and approximately 60% of this surveyed population exhibited β_2_-MG concentration over 300 µg/g creatinine. 

Cadmium daily intake for the Mae Sot population was estimated by multiplying cadmium rice concentration (Rice-Cd) with an average daily rice intake of the Mae Sot population. A survey of 151 paddy fields yielded approximately 700,000 kg (Mae Tao = 68 fields, Mae Ku = 38 fields and Phra Tad Pha Dang = 45 fields). Rice-Cd was derived from a screening survey of Cd in rice production in 2009/2010 by the Mae Sot Agricultural Extension Office (unpublished). The average daily rice consumption was obtained from the survey in the Mae Sot area, which equaled 0.3 kg/day [8]. We made a further assumption that the population in the Mae Sot area consumes locally grown rice so that Cd exposure levels for each subject would correspond to Cd content in rice grown in their area. 

**Figure 3:**
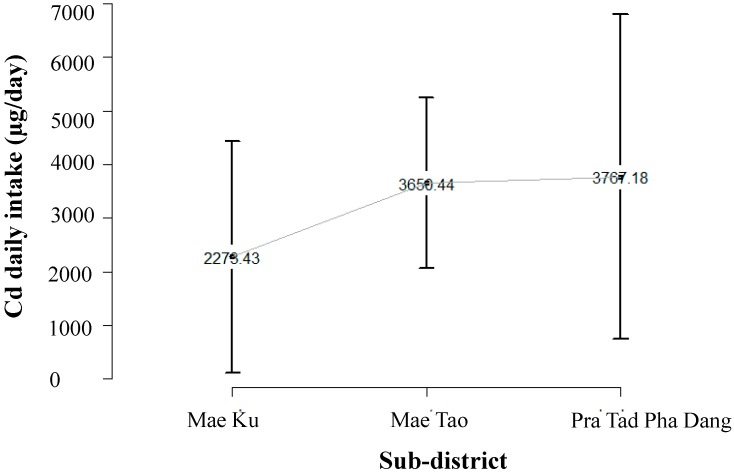
Distribution of cadmium (Cd) daily intake in surveyed paddy fields in Mae Sot areas: paddy fields in Mae Ku (mean = 2273 µg/day, 95% CI = 115–4430)), Mae Tao (mean = 3650 µg /day, 95% CI = 2060–5241) and Pra Tad Pha Dang (mean = 3767 µg/day, 95% CI = 736–6799).

### 2.3. Literature Review to Identify Risk of Cadmium Related Diseases

Health outcomes associated with cadmium exposure in the Mae Sot area were investigated through an extensive review on cadmium toxicity, environmental health studies, and environmental epidemiological studies with the focus on the follow-up studies of the health status of the population living in the cadmium contaminated area [2,25,26].

### 2.4. Additional DALYs Attributable to Cadmium Exposure

By the conventional approach, the DALYs attributable to cadmium are a product of population attributable fraction (PAF) multiplied by total DALYs for a specific disease (*DALYs attributable to Cd* = *PAF × Total DALYs*) [24]. Normally, total DALY parameters equal to national or local DALYs that occurred from all risk factors for specific population. However, it is not possible to estimate total DALYs for this study due to incomplete epidemiological information of Cd-related diseases for this small set of population. Due to this limitation, we opted to present a health impact of living in Mae Sot area through add-DALYs. We assume that Mae Sot residents would originally have total DALYs similar to the general Thai population if they were not exposed to cadmium. Exposure to cadmium would cause an increase in Cd-related disease cases in this area, which eventually can lead to an increase in DALYs of this population. This assumption follows the concept of PAF; therefore, in this study we chose to calculate the PAF value to represent the proportion of disease burden attributable to cadmium exposure [27], and call this “additional DALYs”. Equation (1) was used to calculate PAF for each disease [23,28].

(1) $${\text{PAF }}_{\text{total}}=\overset{\text{k}}{\underset{\text{i}=1}{\sum }}\frac{{\text{PF}}_{\text{i }}\left({\text{Risk}}_{\text{i }}-1\right)}{1+{\text{PF}}_{\text{i }}\left({\text{Risk}}_{\text{i }}-1\right)}$$

PAF _total_: Total population attributable fraction;
PF_i_: Population fraction at exposure level *i*;
Risk_i_: Risk of disease (OR, RR or HR) at exposure level *i*.

**Figure 4:**
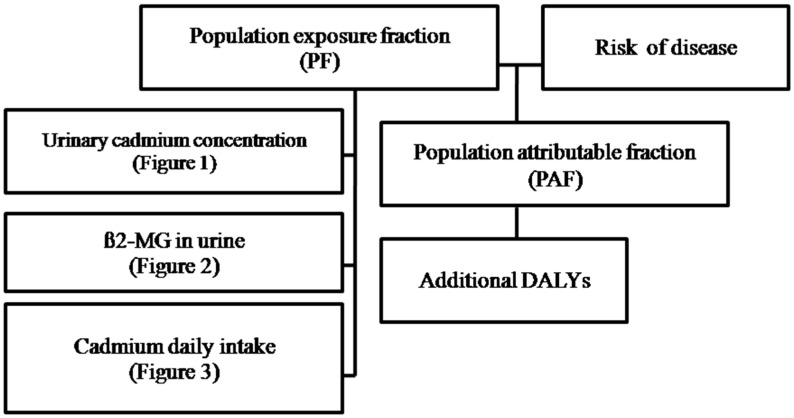
Diagram summarizes the process of additional DALYs attributable to cadmium estimation in Mae Sot area.

We extracted the population exposure fraction (PF) in the Mae Sot area using population exposure distribution data obtained from different types of population exposure data illustrated in Figure 1, Figure 2 and Figure 3. Estimated risk information for diseases in this study is shown in Table 1, Table 2 and Table 3 (Risk of disease in Figure 4). The flow of the studies and data required for each calculation steps is shown in Figure 4.

**Table 1:** Selected studies with U-Cd level and risk of health outcomes for population attributable fraction (PAF) estimation in the Mae Sot area.

Studies	Age (Years)/Gender	Health Outcome	Mean of Cadmium Exposure (µg/g Cr) (95%CI)	Cadmium Exposure Level (µg/g Cr)	Risk of Disease (95%CI)
1.US NHANES; Case control 1988–1994 *n* = 750 cases/6811 controls [29]	30–90/All	Osteoporosis	1.16	0–0.99 1.00–1.99 ≥2.00	Odds ratio 1.00 1.27 (0.88–1.84) 2.59 (1.15–5.83)
2.Swedish mammography cohort (general population) 1997–2009 *n* = 2688/395 cases [30]	56–69/female	Osteoporosis	0.29 (0.14–0.64)	<0.50 0.50–0.75 ≥0.75	Odds ratio Femoral neck 1.00
					2.09 (1.12–3.93)
					3.47 (1.46–8.23)
					Hip or spine
					1.00
					1.27 (0.75–2.14)
					4.24 (1.99–9.04)
3.A 22-year follow-up study in Cd-polluted area in Japan (Kakehashi River) 1981–2003 *n* = 3119/46 cases [31]	≥50/All	Nephritis/nephrosis	Male 4.6 (4.4–4.7) Female 7.2 (7.0–7.4)	<3.0 3.0–4.9 5.0–9.9 ≥10.0	Hazard ratio
					Male
					1.0
					2.7 (0.5–13.2)
					4.4 (1.0–19.9)
					3.4 (0.6–20.5)
					Female
					1.0
					0.7 (0.0–12.4)
					1.4 (0.2–13.1)
					3.1 (0.4–26.2)
4. A 22-year follow-up study in Cd-polluted area in Japan (Kakehashi River) 1981–2003 *n* = 3119/156 cases [31]	≥50/female	Cardiovascular disease	7.2 (7.0–7.4)	<3.0 3.0–4.9 5.0–9.9 ≥10.0	Hazard ratio 1.0
					2.1 (0.9–4.7)
					2.3 (1.1–4.9)
					2.4 (1.1–5.1)
5. A 22-year follow-up study in Cd-polluted area in Japan (Kakehashi River) 1981–2003 *n* = 3119/115 cases [31]	≥50/female	Cerebrovascular disease	7.2 (7.0–7.4)	<3.0 3.0–4.9 5.0–9.9 ≥10.0	Hazard ratio 1.0
					3.0 (0.9–10.5)
					4.3 (1.4–14.0)
					3.6 (1.1–11.9)

Abbreviations: US NHANES = the United States National Health and Nutrition Examination Survey.

**Table 2:** Selected studies with β_2_-MG in urine and risk of health outcome for PAF estimation in Mae Sot area.

Studies	Age (Years)/Gender	Health Outcome	Cadmium Exposure Level (µg/g Cr)	Risk of Disease (95%CI)
1. A 15 year follow-up study in Cd-polluted area in Japan (Kakehashi River) 1981–1996 *n* = 3178 [32]	≥50/All	Nephrosis/Nephritis	<300 300–1000 1000–10,000 ≥10,000	Hazard ratio
				Male
				1.00
				2.44 (0.53–11.2)
				5.67 (1.47–12.8)
				18.15 (4.24–77.6)
				Female
				1.00
				5.43 (0.48–61.5)
				6.94 (0.59–81.7)
				54.98 (5.41–558.5)
2. A 15 year follow-up study in Cd-polluted area in Japan (Kakehashi River) 1981–1996 *n* = 3178 [32]	≥50/All	Heart failure	<300 300–1000 1000–10,000 ≥10,000	Hazard ratio
				Male
				1.00
				0.88 (0.41–1.89)
				1.45 (0.74–2.84)
				3.69 (1.62–8.39)
				Female
				1.00
				1.94 (1.08–3.48)
				3.05 (1.73–5.35)
				3.19 (1.19–5.52)
3. A 15 year follow-up study in Cd-polluted area in Japan (Kakehashi River) 1981–1996 *n* = 3178 [32]	≥50/All	Cerebral infarction	<300 300–1000 1000–10,000 ≥10,000	Hazard ratio
				Male
				1.00
				2.4 (1.15–4.98)
				4.48 (2.29–8.78)
				5.36 (2.04–8.78)
				Female
				1.00
				1.88 (0.82–4.29)
				3.58 (1.71–7.51)
				3.19 (1.29–7.88)

**Table 3:** Selected studies with dietary cadmium level and risk of health outcome for PAF estimation in Mae Sot area.

Studies	Age (Years)/Gender	Health Outcome	Mean of Dietary Cadmium (µg/Day) (±SD)	Cadmium Exposure Level (µg/day)	Risk of Disease (95%CI)
1. Swedish mammography cohort (general population) 1987–2006 *n* = 30,210/378 cases [33]	≥55/female	Post- menopausalendometrial cancer	15	<13.7 13.7–16.0 ≥16.0	Relative risk
					1.00
					1.01 (0.77–1.33)
					1.39 (1.04–1.86)
2. Swedish mammography cohort (general population) 1987–2008 *n* = 55,987/2112 cases [34]	≥55/female	Post-menopausal breast cancer	15 ± 3.2	<13 13–16 >16	Relative risk 1.00
					1.00 (0.90–1.11)
					1.05 (0.95–1.17)
3.The cohort of Swedish Men (general population) 1998–2009 *n* = 41,089/3085 cases [35]	45–79/male	Prostate cancer	19 ± 3	<17 17–20 >20	Relative risk 1.00
					1.18 (1.00–1.40)
					1.29 (1.08–1.53)

## 3. Results

### 3.1. Health Outcomes and Risk Values Associated with Cadmium

Four health outcomes with strong evidence indicating causal relationships between cadmium exposure and outcomes were selected: renal disease, cardiovascular disease, musculoskeletal disorders and cancer [25,36] There is substantial evidence relating cadmium exposure to kidney impairment. These studies measured an association between cadmium exposure and biomarkers for renal dysfunction [25]. We obtained risk information for nephritis/nephrosis, cardiovascular disease, and cerebrovascular disease from the study by Li *et al.*, which covered 3119 residents who were living in the cadmium-polluted Kakehashi River basin and participated in the health survey conducted by Ishikawa Prefecture in 1981–1982. The study participants represented 89% of all inhabitants aged over 50 years of the area during the survey period [31]. For the β_2_-MG study, we included nephrosis/nephritis and two other conditions that could be consequences from renal failure complications: heart failure and cerebral infarction [32,37]. Risk estimates for these conditions were obtained from the 15 year follow-up study of population living in the cadmium polluted Kakahashi River basin in Japan that used β_2_-MG to investigate cadmium associated health outcomes [32].

Cadmium related musculoskeletal and bone outcomes were reported in the cadmium contaminated area. For example, in the Jinzu River Basin area in Japan, “itai-itai” disease and other bone related adverse health outcomes were introduced [38]. Other populations, both living inside and outside cadmium-polluted area, with dietary cadmium exposure also experienced some of the bone diseases such as osteomalacia, osteoporosis, bone fracture or decreased bone mineral density [25]. In this study, we used estimated risk information for osteoporosis from the Swedish mammography cohort and the United States National Health and Nutrition Examination Survey (US NHANES) study [29].

For cancer health outcomes, dietary cadmium exposure was used as an exposure indicator. The risks for prostate cancer, postmenopausal breast cancer and postmenopausal endometrial cancer were obtained from Swedish mammography cohort studies that investigated the associations between cancers and dietary cadmium exposure [33,34,35]. Table 1, Table 2 and Table 3 summarize details of the studies selected for add-DALYs in Mae Sot area.

### 3.2. Additional DALYs Attributable to Cadmium in the Mae Sot Area

Additional DALYs or PAF values are listed in Table 4, Table 5 and Table 6. PF values estimated from the distribution of U-Cd, β_2_-MG and dietary cadmium are listed in Table 4, Table 5 and Table 6 along with Add-DALYs calculated from Equation (1).

**Table 4:** Additional disability adjusted life years (DALYs) estimates from U-Cd in the Mae Sot area.

Cadmium Exposure Level	Male			Female			Both gender		
	Risk	PF	DALY *	Risk	PF	DALY *	Risk	PF	DALY *
1. Nephrosis/nephritis **									
<3	1.00	68	0	1.00	51	0			
3–4.9	2.70	19	25	1.00	26	0			
5–10	4.40	10	26	1.40	18	7			
≥10	3.40	2	6	3.10	6	10			
Total			43			10			
2. Cardiovascular disease **									
<3				1.00	51	0			
3–4.9				2.10	26	22			
5–10				2.30	18	19			
≥10				2.40	6	7			
Total						37			
3. Cerebrovascular disease **									
<3				1.00	51	0			
3–4.9				3.00	26	34			
5–10				4.30	18	37			
≥10				3.60	6	13			
Total						55			
4. Osteoporosis **									
Femoral neck									
<0.5				1.00	2	0			
0.5–0.75				2.09	2	2			
≥0.75				3.47	96	70			
Total						71			
Hip or spine									
<0.5				1.00	2	0			
0.5–0.75				1.27	2	1			
≥0.75				4.24	96	76			
Total						76			
5. Osteoporosis **									
0–0.99							1.00	22	0
1.00–1.99							1.27	28	7
≥2.00							2.59	49	44
Total									46

Abbreviations: Risk = risk of disease; PF = population exposure fraction; DALYs = Disability Adjusted Life years; * DALYs parameter refers to Additional DALYsestimate from urinary cadmium (U-Cd) exposure data of Mae Sot area. Total additional DALYs parameter is equivalent to PAF; ** The range of population’ age for each disease varies as follow: nephrosis/nephitis, cardiovascular disease and cerebrovascular disease (≥50 years), osteoporosis in No.4 (50–69 years), osteoporosis in No. 5 (30–90 years).

The add-DALYs for renal, cardiovascular, cerebrovascular and bone diseases based on U-Cd concentration are as follows (see Table 4): Nephrosis/nephritis (43% for male, 10% for female), cardiovascular disease (37% for female), cerebrovascular disease (55% for female), osteoporosis/femoral neck (71% for female), osteoporosis/hip or spine (76% for female), and osteoporosis (46% for both sexes).

**Table 5:** Additional DALYs estimate from β_2_-MG in urine in the Mae Sot area.

Gender	β_2_-MG (µg/g Cr)	PF	Nephrosis/Nephritis		Heart Failure		Cerebral Infarction	
			Risk	DALY *	Risk	DALY *	Risk	DALY *
Male	<300	0.37	1.00	0	1.00	0	1.00	0
	300–1000	0.17	2.44	20	0.88	0	2.40	19
	1000–10,000	0.29	5.67	58	1.45	12	4.48	50
	≥10,000	0.17	18.15	74	3.69	31	5.36	43
				82		36		67
Female	<300	0.55	1.00	0	1.00	0	1.00	0
	300–1000	0.18	5.43	45	1.94	15	1.88	14
	1000–10,000	0.20	6.94	54	3.05	29	3.58	34
	≥10,000	0.07	54.98	80	3.19	14	3.19	14
				86		43		46

Abbreviations: β_2_-MG = β_2_-microglobulin; PF = population exposure fraction; Risk = risk of disease; DALYs = Disability Adjusted Life years; * DALYs parameter refers to Additional DALYs estimate from cadmium exposure data of Mae Sot area; Total additional DALYs parameter is equivalent to PAF.

**Table 6:** Additional DALYs estimate from dietary cadmium in the Mae Sot area.

Cadmium Exposure Level	Male			Female		
	Risk	PF	DALY *	Risk	PF	DALY *
1. Prostate cancer **						
<17	1.00	0	0			
17–20	1.18	0	0			
> 20	1.29	100	12			
Total			12			
2. Post-menopausal breast cancer *						
<13				1.00	0	0
13–16				1.12	0	0
>16				1.27	100	21
Total						21
3. Post-menopausal endometrial cancer *						
<13.7				1.00	0	0
13.7–16				1.01	0	0
>16				1.39	100	28
Total						28

Abbreviations: Risk = risk of disease; PF = population exposure fraction; DALYs = Disability Adjusted Life years; * DALYs parameter refers to Additional DALYs estimate from dietary cadmium data of Mae Sot area; Total additional DALYs parameter is equivalent to PAF; ** The range of population’ age for each diseases varies as follow; prostate cancer (45–79 years), post-menopausal breast cancer and post-menopausal endometrial cancer (≥55 years).

The add-DALYs for three diseases based on β_2_-MG level are as follows (see Table 5): nephrosis/nephritis (82% for male, 86% for female), heart failure (36% for male, 43% for female) and cerebral infarction (67% for male, 46% for female). 

The add-DALYs for three types of cancer based on dietary cadmium consumption are as follows (see Table 6): prostate cancer (12% for male), post-menopausal breast cancer (21% for female) and post-menopausal endometrial cancer (28% for female).

## 4. Discussion

This study demonstrated the health impact of a population living in cadmium-contaminated land using the additional DALYs approach, which was applied from a method calculating conventional DALYs. The main results suggested that, depending on their exposure level, Mae Sot residents could suffer from several chronic diseases associated with cadmium exposure, such as nephrosis/nephritis, cardiovascular disease, cerebrovascular disease, osteoporosis, and cancer. The magnitude of an increase in DALYs from developing the aforementioned diseases ranged from 10% to 86% depending on the exposure levels. The results were also weighted by the types of exposure biomarker used in the study. For example, for U-Cd concentration, which reflects cadmium body burden, the results were upweighted by U-Cd level of population in the literature used for the risk estimates. Since U-Cd of the Mae Sot population was much higher than the environmental exposure range, applying the risks from the studies in the less contaminated area for risk estimation would result in an underestimation of the PAF. The true risk at the exposure level in our population cannot be accurately estimated. This observation is called “the ceiling effect,” which also applies to the result from external exposure dose data estimated by cadmium content in rice. This effect was not found when studies from the highly cadmium polluted area, where U-Cd was comparable to the concentration in Mae Sot, were used for risk estimation. The third type of indicator used in this study is β_2_-MG that represents a marker of renal dysfunction, which is commonly found in people with chronic exposure to cadmium. Using this biomarker along with the risk estimated from studies with similar exposure concentration, we received more robust results for nephrosis/nephritis and two heart conditions. 

In this study, we chose the health consequences following Cd exposure that were well studied and there was sufficient evidence supporting the association between these diseases and Cd exposure. Since there was no epidemiological study investigating the risk of those diseases developed from Cd exposure for the Mae Sot population, we had to extrapolate the risk values at the Mae Sot population’s exposure level from published studies, which generated some trends in the results of our study. For health conditions using exposure-risk relationship obtained from studies in Cd-contaminated land or case-control studies, approximately 50% of the population examined had U-Cd at the reference level (PF of both conditions were approximately 50%). Similar results were applied to PF values for female population calculated with β_2_-MG data in which the PF values at baseline category accounted for approximately 50%. In contrast, for conditions with general population studies, such as osteoporosis, the high exposure dose of the Mae Sot population leading to the PF value of this population accounted for almost 100% in the highest exposure concentration category, and resulted in very high PAF values (see Equation (1)). We derived similar results when using dietary cadmium as an exposure indicator where PAF values were equal to 100% in the highest exposure category. This ceiling effect prohibited us from estimating the true risk values at Mae Sot exposure concentration, and thereby the reliable PAF values could not be estimated. Since PAF was a product of PF and risk values at different exposure level, the results from studies with ceiling effect were also underestimated because the risk used for PAF calculation was lower than the true one (the mean intake of population in Mae Sot was approximately 100 times higher than the intake from Swedish cohort). As a result, add-DALYs results for these conditions were disregarded from further discussion. However, this ceiling effect was useful to present as supporting evidence to show the severity of cadmium contamination in the Mae Sot area compared to other populations from selected studies. A similar effect was found when using U-Cd as an exposure indicator for osteoporosis data (femoral neck and hip/spine) of which the mean U-Cd of the population in the study was approximately 17 times lower than U-Cd of the Mae Sot population. The exposure distribution for other diseases, when judging by the PF distribution, was well distributed. For the diseases that used studies from the contaminated area, such as nephrosis/nephritis, cardiovascular diseases and cerebrovascular diseases, around 50% or more of population in Mae Sot exhibited cadmium concentration in the baseline categories. This pattern raised a concern regarding the PAF of these diseases that would be underestimated compared to the data of diseases with the ceiling effect, such as osteoporosis that the PF values were shifted to higher exposure categories and thereby corresponding to higher risk values. Add-DALYs for three diseases using β_2_-MG seemed to have the most results obtained from this study. Add-DALYs from nephrosis/nephritis was the highest, which was expected because β_2_-MG was an indicator specifically reflecting renal impairment (impaired tubular re-absorption) [19]. The abundance of this protein provided information regarding renal diseases progression, so that the hazard ratio of these people was also very high (see Table 5), this factor contributed to the add-DALYs over 80% for renal conditions. The other two conditions also caused a significant amount of add-DALYs (but the additional values were less than the values from renal condition), the relationship between patients with renal impairment as a predisposing factor and these diseases was also well established by several toxicological and pathological studies [39,40,41]. The abundance of this biomarker in the Mae Sot population at this high level suggested that these people were also at risk of developing other diseases following renal impairment. These add-DALYs results supported the findings from many health survey studies in the contaminated area, which suggested that at the Mae Sot population’s exposure concentration, the population would be at risk from several cadmium-related health consequences [11,42]. Some studies reported patients suffering from irreversible pathology such as renal impairment that linked to several important chronic health outcomes such as osteoporosis, cardiovascular diseases, hypertension and diabetes [43].

For methodological consideration, we were aware that our study design required secondary data from multiple sources that could generate some uncertainties. However, we selected the data source carefully and interpreted the results with caution from two types of exposure measures used in this study: internal exposure (U-Cd concentration and β_2_-MG in urine) and external exposure doses (daily rice intake). The internal exposure doses were measured from the volunteers, therefore there may be some biases associated with the results, for example, the majority of the population in this study could consist of patients with renal impairment and few healthy subjects. For dietary-Cd intake, we assumed that the whole populations relied on rice grown in their local paddy fields, which may not be the case because it was reported that some people consumed rice grown outside the Mae Sot area to avoid Cd exposure [9,11]. In addition, one might argue that people could be exposed to Cd from other types of food that are contaminated with Cd. We argue that the contribution of Cd-related health outcomes from other food sources is negligible, since data for other types of food was less completed, and rice was the major type of food that people consumed daily. Therefore, our rice survey data well covered the contaminated area.

### Recommendation for Policy Makers

The add-DALYs approach was selected to assess the additional burden of diseases of the Mae Sot population. In this study, we have demonstrated the usefulness of this approach to guide policy decisions. An increase in DALYs reflects the additional fraction of burden of disease that can be eliminated once effective interventions are used to alleviate the problems. For the cadmium situation in Mae Sot, the best solution for this problem is to mitigate the problem by reducing population exposure to cadmium or reducing PF as high cadmium exposure level as shown in other land contamination management strategies of other countries, such as Toyama in Japan [44,45,46].Two major findings from this study show the significance of the problem, percentage of add-DALYs reflecting an increase of disease burden of Mae Sot population compared to the general Thai population and the ceiling effect implying that the exposure dose of Mae Sot people is much higher than populations from other countries with Cd contamination by their standard. The quantification of add-DALYs can be used for priority setting, especially when the data is combined with other useful information such as economic evaluation following health outcome development. 

## 5. Conclusions

This study highlights the significance of environmental cadmium contamination related health problems. To the best of our knowledge, this is the first study using the add-DALYs to investigate the health impact of population living in land contamination problem in Thailand through the healthy life lost parameter. The results indicate that a majority of adverse health outcomes of the population in Mae Sot could increase from exposure to cadmium and also imply that the previous intervention programs employed in this area were inefficient. We hope that this integrated information on the health status of the Mae Sot population can be useful information for policy makers and other relevant authorities for evidence-based policy generation and this approach should be beneficial to other land contamination problems.

## References

[1] Satarug S., Moore M.R. (2004). Adverse health effects of chronic exposure to low-level cadmium in foodstuffs and cigarette smoke. Environ. Health Perspect..

[2] Satarug S., Garrett S.H., Sens M.A., Sens D.A. (2010). Cadmium, environmental exposure, and health outcomes. Environ. Health Perspect..

[3] Nawrot T., Plusquin M., Hogervorst J., Roels H.A., Celis H., Thijs L., Vangronsveld J., Hecke E.V., Staessen J.A. (2006). Environmental exposure to cadmium and risk of cancer: A prospective population-based study. Lancet Oncol..

[4] Bernard A. (2008). Cadmium & its adverse effects on human health. Indian J. Med. Res..

[5] Järup L., Åkesson A. (2009). Current status of cadmium as an environmental health problem. Toxicol. Appl. Pharmacol..

[6] WHO (1992). International Programme on Chemical Safety: Environmental Health Criteria 134. Cadmium.

[7] Bernard A. (2004). Renal dysfunction induced by cadmium: Biomarkers of critical effects. Biometals.

[8] Simmons R., Pongsakul P., Saiyasitpanich D., Klinphoklap S. (2005). Elevated levels of cadmium and zinc in paddy soils and elevated levels of cadmium in rice grain downstream of a zinc mineralized area in Thailand: Implications for public health. Environ. Geochem. Health.

[9] Swaddiwudhipong W., Mahasakpan P., Funkhiew T., Limpatanachote P. (2010). Changes in cadmium exposure among persons living in cadmium-contaminated areas in northwestern Thailand: A five-year follow-up. J. Med. Assoc. Thai..

[10] Swaddiwudhipong W., Limpatanachote P., Mahasakpan P., Krintratun S., Padungtod C. (2007). Cadmium-exposed population in Mae Sot District, Tak Province: 1. Prevalence of high urinary cadmium levels in the adults. J. Med. Assoc. Thai..

[11] Swaddiwudhipong W., Limpatanachote P., Mahasakpan P., Krintratun S., Punta B., Funkhiew T. (2012). Progress in cadmium-related health effects in persons with high environmental exposure in northwestern Thailand: A five-year follow-up. Environ. Res..

[12] Honda R., Swaddiwudhipong W., Nishijo M., Mahasakpan P., Teeyakasem W., Ruangyuttikarn W., Satarug S., Padungtod C., Nakagawa H. (2010). Cadmium induced renal dysfunction among residents of rice farming area downstream from a zinc-mineralized belt in Thailand. Toxicol. Lett..

[13] Limpatanachote P., Swaddiwudhipong W., Mahasakpan P., Krintratun S. (2009). Cadmium-exposed population in Mae Sot District, Tak Province: 2. Prevalence of renal dysfunction in the adults. J. Med. Assoc. Thai..

[14] Limpatanachote P., Swaddiwudhipong W., Nishijo M., Honda R., Mahasakpan P., Nambunmee K., Ruangyuttikarn W. (2010). Cadmium-exposed population in Mae Sot District, Tak Province: 4 bone mineral density in persons with high cadmium exposure. J. Med. Assoc. Thai..

[15] Nambunmee K., Honda R., Nishijo M., Swaddiwudhipong W., Nakagawa H., Ruangyuttikarn W. (2010). Bone resorption acceleration and calcium reabsorption impairment in a Thai population with high cadmium exposure. Toxicol. Mech. Methods.

[16] Swaddiwudhipong W., Limpatanachote P., Nishijo M., Honda R., Mahasakpan P., Krintratun S. (2010). Cadmium-exposed population in Mae Sot district, Tak province: 3. Associations between urinary cadmium and renal dysfunction, hypertension, diabetes, and urinary stones. J. Med. Assoc. Thai..

[17] Swaddiwudhipong W., Mahasakpan P., Limpatanachote P., Krintratun S. (2010). Correlations of urinary cadmium with hypertension and diabetes in persons living in cadmium-contaminated villages in northwestern Thailand: A population study. Environ. Res..

[18] Swaddiwudhipong W., Mahasakpan P., Limpatanachote P., Krintratun S. (2011). An association between urinary cadmium and urinary stone disease in persons living in cadmium-contaminated villages in northwestern Thailand: A population study. Environ. Res..

[19] Teeyakasem W., Nishijo M., Honda R., Satarug S., Swaddiwudhipong W., Ruangyuttikarn W. (2007). Monitoring of cadmium toxicity in a Thai population with high-level environmental exposure. Toxicol. Lett..

[20] Padungtod C., Swaddiwudhipong W. (2003). Health Risk Management for Cadmium Contamination in Thailand: Are Challenges Overcome?. http://www.who.int/ifcs/documents/forums/forum5/thai_padungtod.pdf.

[21] Veerman J.L., Barendregt J.J., Mackenbach J.P. (2005). Quantitative health impact assessment: Current practice and future directions. J. Epidemiol. Community Health.

[22] McCarthy M., Biddulph J.P., Utley M., Ferguson J., Gallivan S. (2002). A health impact assessment model for environmental changes attributable to development projects. J. Epidemiol. Community Health.

[23] Lim S.S., Vos T., Flaxman A.D., Danaei G., Shibuya K., Adair-Rohani H., Amann M., Anderson H.R., Andrews K.G., Aryee M. (2012). A comparative risk assessment of burden of disease and injury attributable to 67 risk factors and risk factor clusters in 21 regions, 1990–2010: A systematic analysis for the global burden of disease study 2010. Lancet.

[24] Murray C.J., Ezzati M., Lopez A.D., Rodgers A., Hoorn S.V. (2003). Comparative quantification of health risks conceptual framework and methodological issues. Popul. Health Metr..

[25] Faroon O., Ashizawa A., Wright S., Tucker P., Jenkins K., Ingerman L., Rudisill C. (2012). Toxicological Profile for Cadmium.

[26] Cho Y.A., Kim J., Woo H.D., Kang M. (2013). Dietary cadmium intake and the risk of cancer: A meta-analysis. PLoS ONE.

[27] Levine B. (2007). What does the population attributable fraction mean?. Prev. Chronic Dis..

[28] Hanley J.A. (2001). A heuristic approach to the formulas for population attributable fraction. J. Epidemiol. Community Health.

[29] Wu Q., Magnus J., Hentz J. (2010). Urinary cadmium, osteopenia, and osteoporosis in the US population. Osteoporos. Int..

[30] Engström A., Michaëlsson K., Suwazono Y., Wolk A., Vahter M., Åkesson A. (2011). Long-term cadmium exposure and the association with bone mineral density and fractures in a population-based study among women. J. Bone Miner. Res..

[31] Li Q., Nishijo M., Nakagawa H., Morikawa Y., Sakurai M., Nakamura K., Kido T, Nogawa K., Min D. (2011). Relationship between urinary cadmium and mortality in habitants of a cadmium-polluted area: A 22-year follow-up study in Japan. Chin. Med. J (Engl.).

[32] Nishijo M., Morikawa Y., Nakagawa H., Tawara K., Miura K., Kido T., Ikawa A., Kobayashi E., Nogawa K. (2006). Causes of death and renal tubular dysfunction in residents exposed to cadmium in the environment. Occup. Environ. Med..

[33] Akesson A., Julin B., Wolk A. (2008). Long-term dietary cadmium intake and postmenopausal endometrial cancer incidence: A population-based prospective cohort study. Cancer Res..

[34] Julin B., Wolk A., Bergkvist L., Bottai M., Akesson A. (2012). Dietary cadmium exposure and risk of postmenopausal breast cancer: A population-based prospective cohort study. Cancer Res..

[35] Julin B., Wolk A., Johansson J.E., Andersson S.O., Andren O., Akesson A. (2012). Dietary cadmium exposure and prostate cancer incidence: A population-based prospective cohort study. Br. J. Cancer.

[36] Tellez-Plaza M., Jones M.R., Dominguez-Lucas A., Guallar E., Navas-Acien A. (2013). Cadmium exposure and clinical cardiovascular disease: A systematic review. Curr. Atherosclero. Rep..

[37] Nishijo M., Nakagawa H., Morikawa Y., Kuriwaki J., Katsuyuki M., Kido T., Nogawa K. (2004). Mortality in a cadmium polluted area in Japan. Biometals.

[38] Kobayashi E., Suwazono Y., Dochi M., Honda R., Kido T. (2009). Influence of consumption of cadmium-polluted rice or Jinzu River water on occurrence of renal tubular dysfunction and/or Itai-itai disease. Biol. Trace Elem. Res..

[39] Salam S.N., Eastell R., Khwaja A. (2014). Fragility fractures and osteoporosis in CKD: Pathophysiology and diagnostic methods. Am. J. Kidney Dis..

[40] Toyoda K., Ninomiya T. (2014). Stroke and cerebrovascular diseases in patients with chronic kidney disease. Lancet Neurol..

[41] Noonan C.W., Sarasua S.M., Campagna D., Kathman S.J., Lybarger J.A., Mueller P.W. (2002). Effects of exposure to low levels of environmental cadmium on renal biomarkers. Environ. Health Perspect..

[42] Kobayashi E., Okubo Y., Suwazono Y., Kido T., Nogawa K. (2002). Dose–response relationship between total cadmium intake calculated from the cadmium concentration in rice collected from each household of farmers and renal dysfunction in inhabitants of the Jinzu River basin, Japan. J. Appl. Toxicol..

[43] Maruzeni S., Nishijo M., Nakamura K., Morikawa Y., Sakurai M., Nakashima M., Kido T., Okamoto R., Nogawa K., Suwazono Y., Nakagawa H. (2014). Mortality and causes of deaths of inhabitants with renal dysfunction induced by cadmium exposure of the polluted Jinzu River basin, Toyama, Japan; a 26-year follow-up. Environ. Health.

[44] Kaji M. (2012). Role of experts and public participation in pollution control: The case of Itai-itai disease in Japan. Ethics Sci. Environ. Polit..

[45] Ferguson C.C. (1999). Assessing risks from contaminated sites: Policy and practice in 16 European countries. Land Contamin. Reclam..

[46] Besante J., Niforatos J., Mousavi A. (2011). Cadmium in Rice: Disease and Social Considerations. Environ. Forensic..

